# Complete genome sequence of *Segniliparus rotundus* type strain (CDC 1076^T^)

**DOI:** 10.4056/sigs.791633

**Published:** 2010-03-30

**Authors:** Johannes Sikorski, Alla Lapidus, Alex Copeland, Monica Misra, Tijana Glavina Del Rio, Matt Nolan, Susan Lucas, Feng Chen, Hope Tice, Jan-Fang Cheng, Marlen Jando, Susanne Schneider, David Bruce, Lynne Goodwin, Sam Pitluck, Konstantinos Liolios, Natalia Mikhailova, Amrita Pati, Natalia Ivanova, Konstantinos Mavromatis, Amy Chen, Krishna Palaniappan, Olga Chertkov, Miriam Land, Loren Hauser, Yun-Juan Chang, Cynthia D. Jeffries, Thomas Brettin, John C. Detter, Cliff Han, Manfred Rohde, Markus Göker, Jim Bristow, Jonathan A. Eisen, Victor Markowitz, Philip Hugenholtz, Nikos C. Kyrpides, Hans-Peter Klenk

**Affiliations:** 1 DSMZ - German Collection of Microorganisms and Cell Cultures GmbH, Braunschweig, Germany; 2 DOE Joint Genome Institute, Walnut Creek, California, USA; 3 Los Alamos National Laboratory, Bioscience Division, Los Alamos, New Mexico, USA; 4 Biological Data Management and Technology Center, Lawrence Berkeley National Laboratory, Berkeley, California, USA; 5 Lawrence Livermore National Laboratory, Livermore, California, USA; 6 Oak Ridge National Laboratory, Oak Ridge, Tennessee, USA; 7 HZI - Helmholtz Centre for Infection Research, Braunschweig, Germany; 8 University of California Davis Genome Center, Davis, California, USA

**Keywords:** aerobic, non-sporeforming, novel mycolic acid, opportunistic pathogen, *Corynebacterineae*, GEBA

## Abstract

*Segniliparus rotundus* Butler 2005 is the type species of the genus *Segniliparus*, which is currently the only genus in the corynebacterial family *Segniliparaceae*. This family is of large interest because of a novel late-emerging genus-specific mycolate pattern. The type strain has been isolated from human sputum and is probably an opportunistic pathogen. Here we describe the features of this organism, together with the complete genome sequence and annotation. This is the first completed genome sequence of the family *Segniliparaceae*. The 3,157,527 bp long genome with its 3,081 protein-coding and 52 RNA genes is part of the *** G****enomic* *** E****ncyclopedia of* *** B****acteria and* *** A****rchaea * project.

## Introduction

Strain CDC 1076^T^ (= DSM 44985 = ATCC BAA-972 = JCM 13578) is the type strain of the species *Segniliparus rotundus* [1], which is the type species of the genus *Segniliparus*. Besides *S. rotundus*, the genus *Segniliparus* contains currently only one additional species: *S. rugosus* at present[1]. *Segniliparus* is currently the only genus in the family *Segniliparaceae*. The generic name of the genus derives from the Latin word ‘segnis’, meaning ‘slow’, and the Greek word ‘liparos’, fat/fatty, meaning ‘one with slow fats’, to indicate the possession of slow reacting fatty acids, i.e., late eluting mycolic acids detected with HPLC [1]. The species name is derived from the Latin word ‘rotundus’, rounded, referring to the smooth, round-domed colony forms [1]. Strain CDC 1076^T^ was isolated from human sputum in Tennessee, USA [1]. Currently, only one additional strain of the species, CDC 413 (with identical 16S rRNA gene sequence), is known, which has been isolated from the human nasal region in Missouri, USA [1]. The 16S rRNA gene sequence of the type strain for the second species in the genus, *S. rugosus* [1], differs by only 1.1% from that of strain CDC 1076^T^. *S. rugosus* strains have been isolated from patients with cystic fibrosis in Australia and most probably USA [2,3], suggesting that *S. rotundus* could also be an opportunistic pathogen. The next closest relatives of *S. rotundus* outside the genus are the members of the genus *Rhodococcus*, which share 93.3 to 94.8% 16S rRNA genes sequence similarity with strain CDC 1076^T^ [4]. Environmental screens and metagenomic surveys did not detected a single phylotype with more than 90-92% 16S rRNA gene sequence similarity, indicating a rather limited ecological distribution of the members of the genus *Segniliparus* (status February 2010). Here we present a summary classification and a set of features for *S. rotundus* CDC 1076^T^, together with the description of the complete genomic sequencing and annotation.

## Classification and features

**Figure 1:**
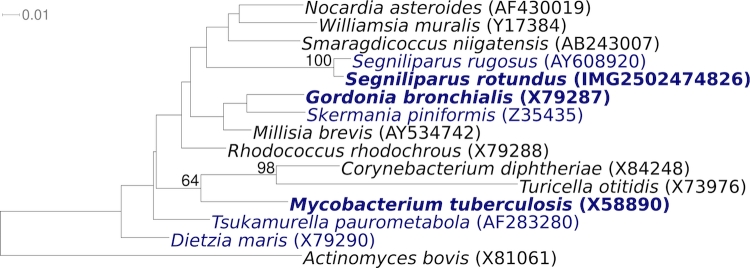
Phylogenetic tree highlighting the position of *S. rotundus* CDC 1076^T^ relative to the other type strains within the suborder *Corynebacterineae*. The tree was inferred from 1,436 aligned characters [5,6] of the 16S rRNA gene sequence under the maximum likelihood criterion [7] and rooted with the type strains of the order *Actinomycetales*. The branches are scaled in terms of the expected number of substitutions per site. Numbers above branches are support values from 350 bootstrap replicates [8] if larger than 60%. Lineages with type strain genome sequencing projects registered in GOLD [9] are shown in blue, published genomes in bold [10,11].

Figure 1 shows the phylogenetic neighborhood of for *S. rotundus* CDC 1076^T^ in a 16S rRNA based tree. The sequence of the sole 16S rRNA gene in the genome is identical with the previously published 16S rRNA sequence generated from DSM 44985 (AY608918).

**Figure 2:**
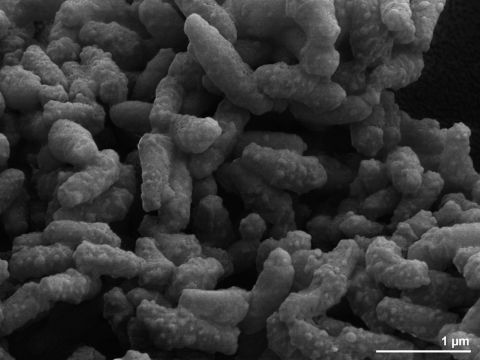
Scanning electron micrograph of *S. rotundus* CDC 1076^T^

CDC 1076^T^ cells are short rods with 0.4µm width by 1.0-1.3 µm length (Table 1 and Figure 2), forming round, smooth, dense and domed colonies [1]. Occasionally, v-forms are produced, but no true branching, mycelium, or spores have been reported. The colonies are non-pigmented, non-photochromogenic and do not produce a diagnostic odor [1]. It is negative for arylsulfatase after three days but positive after 14 days. Strain CDC 1076^T^ does not grow on MacConkey agar, is weakly positive for iron uptake, Tween opacity and Tween hydrolysis, but negative for nitrate and tellurite reduction and for growth in lysozyme (21 days) [1]. Strain CDC 1076^T^ does not produce niacin and develops bubbles in the semi-quantitative catalase test [1]. Using the API CORYNE test kit, strain CDC 1076^T^ is positive for β-glucosidase and pyrazinamidase activities and negative for alkaline phosphatase, β-galactosidase, β-glucuronidase, α-glucosidase, N-acetyl-β-glucosaminidase and pyrrolidonyl arylamidase activity at 33°C [1]. Strain CDC 1076^T^ is susceptible to amikacin, cefoxitan, clarithromycin, ciprofloxacin, doxycycline, imipenem and sulfamethoxazole at or below the respective MIC breakpoints but intermediate to tobramycin [1]. Glucose, maltose, D-fructose and trehalose are used as carbon source for growth with acid production, but not adonitol, L-arabinose, cellobiose, dulcitol, i-erythritol, galactose, i-myo-inositol, lactose, mannose, melibiose, raffinose, L-rhamnose, salicin, D-mannitol, D-sorbitol and sodium citrate [1]. Strain CDC 1076^T^ hydrolyzes urea but not acetamide, adenine, casein, citrate, aesculin, hypoxanthine, tyrosine and xanthine [1].

**Table 1:** Classification and general features of *S. rotundus* CDC 1076^T^ according to the MIGS recommendations [12]

**MIGS ID**	**Property**	**Term**	**Evidence code**
	Current classification	Domain *Bacteria*	TAS [13]
		Phylum *Actinobacteria*	TAS [14]
		Class *Actinobacteria*	TAS [15]
		Subclass *Actinobacteridae*	TAS [15]
		Order *Actinomycetales*	TAS [15]
		Suborder *Corynebacterineae*	TAS [15]
		Family *Segniliparaceae*	TAS [1]
		Genus *Segniliparus*	TAS [1]
		Species *Segniliparus rotundus*	TAS [1]
		Type strain CDC 1076	TAS [1]
	Gram stain	Gram-negative	NAS
	Cell shape	short rods	TAS [1]
	Motility	nonmotile	TAS [1]
	Sporulation	non-sporulating	TAS [1]
	Temperature range	mesophile, 28°C - 37°C	TAS [1]
	Optimum temperature	33°C	TAS [1]
	Salinity	not determined	
MIGS-22	Oxygen requirement	aerobic	TAS [1]
	Carbon source	glucose, maltose, D-fructose, trehalose	TAS [1]
	Energy source	chemoorganotroph	TAS [1]
MIGS-6	Habitat	unknown, but probably host associated	TAS [1]
MIGS-15	Biotic relationship	unknown	
MIGS-14	Pathogenicity	most probably opportunistic pathogen	TAS [1-3]
	Biosafety level	2	TAS [16]
	Isolation	human sputum	TAS [1]
MIGS-4	Geographic location	Tennessee, USA	TAS [1]
MIGS-5	Sample collection time	2005 or before	TAS [1]
MIGS-4.1 MIGS-4.2	Latitude Longitude	unknown	
MIGS-4.3	Depth	unknown	
MIGS-4.4	Altitude	unknown	

Evidence codes - IDA: Inferred from Direct Assay (first time in publication); TAS: Traceable Author Statement (i.e., a direct report exists in the literature); NAS: Non-traceable Author Statement (i.e., not directly observed for the living, isolated sample, but based on a generally accepted property for the species, or anecdotal evidence). These evidence codes are from of the Gene Ontology project [17]. If the evidence code is IDA, then the property was directly observed for a live isolate by one of the authors or an expert mentioned in the acknowledgements.

### Chemotaxonomy

The cell wall of strain CDC 1076^T^ contains mycolic acids and *meso*-diaminopimelic acid [1]. The mycolic acid HPLC pattern is a triple cluster of contiguous eluting peaks starting at approx. 6.0 min and ending with the last peak co-eluting with the internal standard. The TLC mycolic acid pattern reveals α^+^- and α-mycolates [1]. The fatty acids composition of the strain is dominated by straight-chain saturated acids such as the taxon-specific C_10:0_ (21.0%), C_16:0_ (18.5%), C_14:0_ (15.3%), 10-methyl-C_18:0_ (7.4%, tuberculostearic acid), C_20:0_ (4.9%), C_12:0_ (2.4%), C_18:0_ (1.9%), with some by straight-chain desaturated acids, C_18:1 cis_ (15.1%) and C16:1ω9t (9.7%); (personal communication with R.M. Kroppenstedt). Quinones are mainly MK 8(H_4_) and MK 8(H_2_) with some MK 8(H_6_) and traces of MK 9(H_2_) (R.M. Kroppenstedt, personal communication).

## Genome sequencing and annotation

### Genome project history

This organism was selected for sequencing on the basis of its phylogenetic position, and is part of the *** G****enomic* *** E****ncyclopedia of* *** B****acteria and* *** A****rchaea * project [18]. The genome project is deposited in the Genome OnLine Database [9] and the complete genome sequence is deposited in GenBank. Sequencing, finishing and annotation were performed by the DOE Joint Genome Institute (JGI). A summary of the project information is shown in Table 2.

**Table 2:** Genome sequencing project information

**MIGS ID**	**Property**	**Term**
MIGS-31	Finishing quality	Finished
MIGS-28	Libraries used	Two genomic 454 libraries: one standard and one 4kb PE; one Illumina shotgun library
MIGS-29	Sequencing platforms	454 GS FLX Titanium, Illumina GAii
MIGS-31.2	Sequencing coverage	58.1× 454 pyrosequence, 73.3× Illumina
MIGS-30	Assemblers	Newbler version 12.0.1 PreRelease 3/30/2009.1.02.15, Velvet, phrap
MIGS-32	Gene calling method	Prodigal
	INSDC ID	CP001958
	GenBank Date of Release	not yet
	GOLD ID	Gc01232
	NCBI project ID	37711
	Database: IMG-GEBA	2502422312
MIGS-13	Source material identifier	DSM 44985
	Project relevance	Tree of Life, GEBA

### Growth conditions and DNA isolation

*S. rotundus* CDC 1076^T^, DSM 44985, was grown in DSMZ medium 645 (Middlebrook Medium) [19] at 28°C. DNA was isolated from 1-1.5 g of cell paste using Qiagen Genomic 500 DNA Kit (Qiagen, Hilden, Germany) with lysis modification LALMP according to Wu *et al*. [18].

### Genome sequencing and assembly

The genome was sequenced using a combination of Illumina and 454 technologies [20]. An Illumina GAii shotgun library with reads of 443 Mb, a 454 Titanium draft library with average read length of 304 bases, and a paired-end 454 library with average insert size of 4 Kb were generated for this genome. All general aspects of library construction and sequencing can be found at http://www.jgi.doe.gov/. Illumina sequencing data was assembled with VELVET [21] and the consensus sequences were shredded into 1.5 kb overlapped fake reads and assembled together with the 454 data. Draft assemblies were based on 183 Mb 454 data, and 454 paired-end data. Newbler parameters are -consed -a 50 -l 350 -g -m -ml 20. The initial assembly contained 26 contigs in one scaffold. We converted the initial 454 assembly into a phrap assembly by making fake reads from the consensus, collecting the read pairs in the 454 paired-end library. The Phred/Phrap/Consed software package (www.phrap.com) was used for sequence assembly and quality assessment [18] in the following finishing process. After the shotgun stage, reads were assembled with parallel phrap (High Performance Software, LLC). Possible mis-assemblies were corrected with gapResolution (unpublished, http://www.jgi.doe.gov/), Dupfinisher [22], or sequencing cloned bridging PCR fragments with subcloning or transposon bombing (Epicentre Biotechnologies, Madison, WI). Gaps between contigs were closed by editing in Consed, by PCR and by Bubble PCR (J-F Cheng, unpublished) primer walks. A total of 108 additional reactions were necessary to close gaps and to raise the quality of the finished sequence. The completed genome sequences had an error rate less than one in 100,000 bp.

### Genome annotation

Genes were identified using Prodigal [23] as part of the Oak Ridge National Laboratory genome annotation pipeline, followed by a round of manual curation using the JGI GenePRIMP pipeline [24]. The predicted CDSs were translated and used to search the National Center for Biotechnology In-formation (NCBI) nonredundant database, UniProt, TIGRFam, Pfam, PRIAM, KEGG, COG, and InterPro databases. Additional gene prediction analysis and manual functional annotation was performed within the Integrated Microbial Genomes Expert Review (IMG-ER) platform [25].

## Genome properties

**Figure 3:**
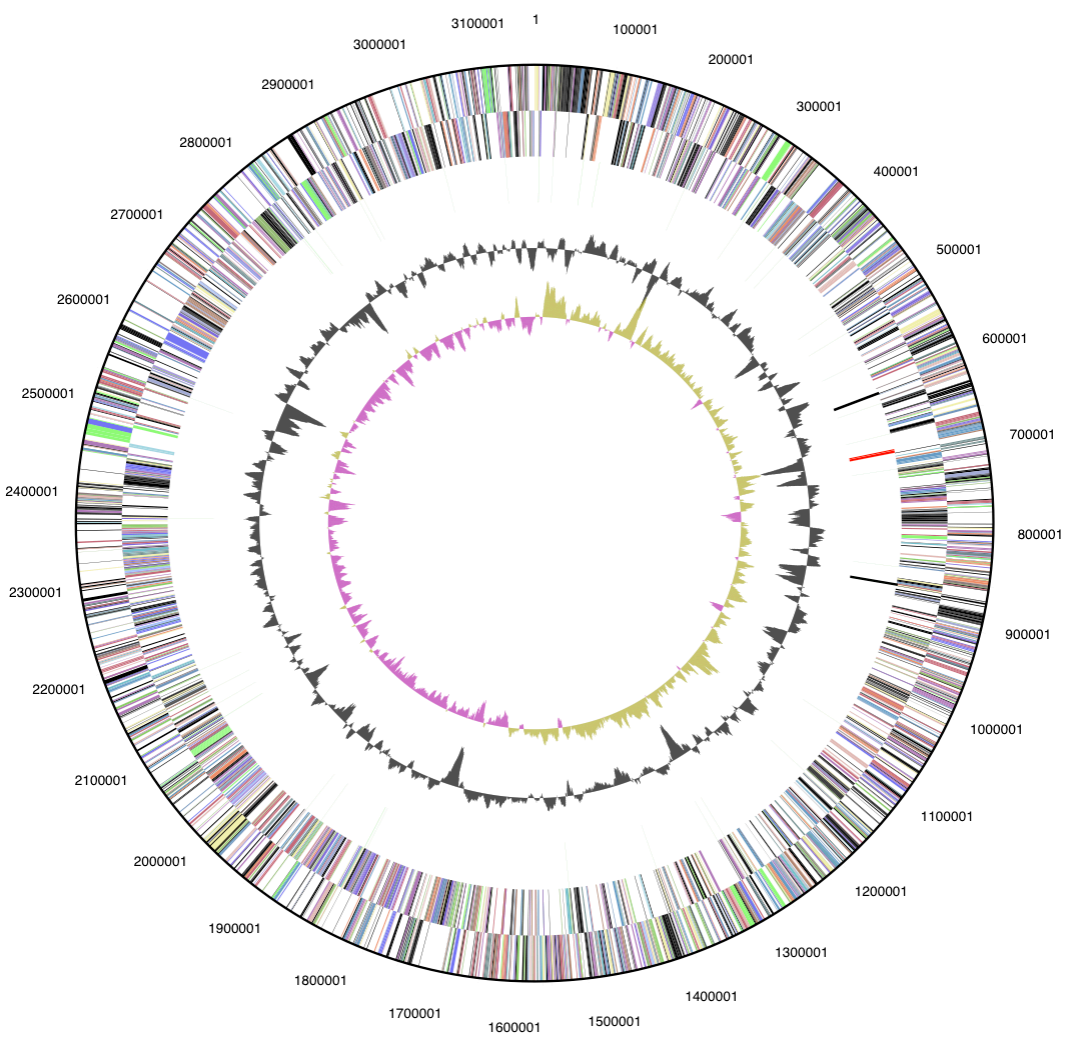
Graphical circular map of the genome. From outside to the center: Genes on forward strand (color by COG categories), Genes on reverse strand (color by COG categories), RNA genes (tRNAs green, rRNAs red, other RNAs black), GC content, GC skew.

The genome consists of a 3,157,527 bp long chromosome (Table 3 and Figure 3). Of the 3,133 genes predicted, 3,081 were protein-coding genes, and 52 RNAs; 75 pseudogenes were also identified. The majority of the protein-coding genes (63.0%) were assigned with a putative function while those remaining were annotated as hypothetical proteins. The distribution of genes into COGs functional categories is presented in Table 4.

**Table 3:** Genome Statistics

**Attribute**	**Value**	**% of Total**
Genome size (bp)	3,157,527	100.00%
DNA coding region (bp)	2,914,227	92.29%
DNA G+C content (bp)	2,108,953	66.79%
Number of replicons	1	
Extrachromosomal elements	0	
Total genes	3,133	100.00%
RNA genes	52	1.66%
rRNA operons	1	
Protein-coding genes	3,081	98.34%
Pseudo genes	75	2.39%
Genes with function prediction	1,974	63.01%
Genes in paralog clusters	442	14.11%
Genes assigned to COGs	1,861	59.40%
Genes assigned Pfam domains	2,097	66.93%
Genes with signal peptides	848	27.07%
Genes with transmembrane helices	671	21.42%
CRISPR repeats	0	

**Table 4:** Number of genes associated with the general COG functional categories

**Code**	**value**	**%age**	**Description**
J	134	4.3	Translation, ribosomal structure and biogenesis
A	1	0.0	RNA processing and modification
K	126	4.1	Transcription
L	114	3.7	Replication, recombination and repair
B	0	0.0	Chromatin structure and dynamics
D	22	0.7	Cell cycle control, cell division, chromosome partitioning
Y	0	0.0	Nuclear structure
V	20	0.7	Defense mechanisms
T	58	1.9	Signal transduction mechanisms
M	97	3.1	Cell wall/membrane biogenesis
N	4	0.1	Cell motility
Z	0	0.0	Cytoskeleton
W	0	0.0	Extracellular structures
U	23	0.7	Intracellular trafficking, secretion, and vesicular transport
O	82	2.7	Posttranslational modification, protein turnover, chaperones
C	141	4.6	Energy production and conversion
G	125	4.1	Carbohydrate transport and metabolism
E	209	6.8	Amino acid transport and metabolism
F	77	2.5	Nucleotide transport and metabolism
H	116	3.8	Coenzyme transport and metabolism
I	117	3.8	Lipid transport and metabolism
P	103	3.3	Inorganic ion transport and metabolism
Q	85	2.8	Secondary metabolites biosynthesis, transport and catabolism
R	247	8.0	General function prediction only
S	149	4.8	Function unknown
-	1,272	41.3	Not in COGs
